# Cellular and humoral immune responses in sheep vaccinated with candidate antigens MAP2698c and MAP3567 from *Mycobacterium avium* subspecies *paratuberculosis*

**DOI:** 10.3389/fcimb.2014.00093

**Published:** 2014-07-16

**Authors:** Ratna B. Gurung, Auriol C. Purdie, Richard J. Whittington, Douglas J. Begg

**Affiliations:** Faculty of Veterinary Science, The University of SydneyCamden, NSW, Australia

**Keywords:** adjuvant, antibody, antigen, ELISA, paratuberculosis, sheep

## Abstract

Control of Johne's disease, caused by *Mycobacterium avium* subspecies *paratuberculosis* (MAP) in ruminants using commercially available vaccine reduces production losses, mortality, fecal shedding and histopathological lesions but does not provide complete protection from infection and interferes with serological diagnosis of Johne's disease and bovine tuberculosis. At this time no recombinant antigens have been found to provide superior protection compared to whole killed or live-attenuated MAP vaccines. Therefore, there is a need to evaluate more candidate MAP antigens. In this study recombinant MAP antigens MAP2698c and MAP3567 were formulated with four different MONTANIDE™ (ISA 50V2, 61VG, 71VG, and 201VG) adjuvants and evaluated for their ability to produce specific immune responses in vaccinated sheep. The cellular immune response was measured with an interferon-gamma (IFN-γ) release assay and the humoral immune response was measured by antibody detection enzyme linked immunosorbent assay. Recombinant vaccine formulation with the antigen MAP2698c and MONTANIDE™ ISA 201VG adjuvant produced strong whole-MAP as well as MAP2698c-specific IFN-γ responses in a high proportion of the vaccinated sheep. The formulation caused less severe injection site lesions in comparison to other formulations. The findings from this study suggest that the MAP2698c + 201VG should be evaluated in a challenge trial to determine the efficacy of this vaccine candidate.

## Introduction

Johne's disease caused by *Mycobacterium avium* subspecies *paratuberculosis* (MAP), is an economically significant disease of ruminant species particularly cattle and sheep (Ott et al., 1999; Morris et al., 2006). MAP vaccines used in livestock contain heat-killed (Gudair® 316F strain, Mycopar strain 18, ID-Lelystad and 5889 Bergey) or live modified (Neoparasec-strain 316F and OSLO-strain 316F/2E) MAP cells Rosseels and Huygen (2008). Vaccination using a currently available commercial vaccine reduces production loss, mortality, histopathological lesions (Wentink et al., 1994; Griffin et al., 2009), bacterial shedding in feces (Kormendy, 1994; Juste et al., 2009; Alonso-Hearn et al., 2012) and extends the average life of vaccinated animals but does not provide complete protection from infection (Reddacliff et al., 2006; Windsor, 2006). Furthermore, the use of killed or live-attenuated vaccine is limited mainly to sheep due to the cross reaction it produces with the immunological diagnosis of bovine tuberculosis in cattle (Stringer et al., 2011). Potential inadvertent self-injection by the handler with the commercial vaccines is also a concern due to severe injection site reactions (Windsor et al., 2005; Windsor, 2006). Recombinant MAP vaccines should have various merits over killed or attenuated vaccines in terms of antigen production and human safety. The most commonly evaluated recombinant proteins have been Hsp 70 (Koets et al., 2006), antigen 85, 74F, SOD, 35 kDa (Chen et al., 2008; Kathaperumal et al., 2008; Park et al., 2008), mpt (Heinzmann et al., 2008), 95 kDa (Bull et al., 2007), P22 (Rigden et al., 2006), 65 kDa (Velaz-Faircloth et al., 1999), and 16.8 kDa (Kadam et al., 2009). Many of the recombinant vaccines were reported to induce strong cellular as well as antibody mediated immune responses (Rigden et al., 2006; Kathaperumal et al., 2008; Roupie et al., 2008). Some of the recombinant vaccines also induced partial protection from infection (Kathaperumal et al., 2009). At this time no recombinant MAP antigens are used in commercial vaccines. Therefore, there is a need to evaluate more MAP antigens to identify potential vaccine candidates.

MAP2698c, a fatty acid dehydrogenase encoded by the *desA2* gene, is an ortholog of Rv1094 of *Mycobacterium tuberculosis* involved in mycobacterial fatty acid metabolism, which is important for maintaining a robust cell wall, intracellular survival, growth and pathogenicity (Dyer et al., 2005). MAP3567 is a surface-exposed hypothetical protein overlapped with cell wall protein (He and De Buck, 2010). Both antigens were reported to be upregulated under *in vitro* stress conditions (Gumber and Whittington, 2009). *In silico* analysis suggested that MAP2698c and MAP3567 proteins contained relatively more T and B cell epitopes than other stress regulated MAP proteins (Gurung et al., 2012a). These proteins were found to be detected by antibodies and induced recall of cell mediated immune responses from MAP infected sheep suggesting that they are also expressed under *in vivo* conditions as they are recognized by the host immune system. Therefore, we are investigating their potential immunogenicity as vaccine antigens.

The aim of this study was to evaluate cellular immune response using interferon-gamma (IFN-γ) release assay as well as humoral immune response using antibody enzyme linked immunosorbent assay (ELISA) in sheep against the recombinant antigens MAP2698c and MAP3567, when they were administered in formulation with four mineral oil based adjuvants from the MONTANIDE ISA range.

## Materials and methods

### Animals

A total of 34 Merino wethers, between 24 and 36 months of age were sourced from a flock in Armidale, New South Wales, Australia and moved to the University of Sydney farms. The source flock was monitored and tested negative for more than 3 consecutive years (MN3) under the Market Assurance Program for sheep (Animal Health Australia). Among the 34 wethers, 32 were randomly divided into eight groups of four animals for vaccination and the remaining two sheep were used as unvaccinated controls. All animal experiments in this study were carried out with approval from the University of Sydney Animal Ethics Committee. During the study the animals were managed under conventional Australian sheep farming conditions by grazing in open paddocks.

### Antigen and adjuvant

Recombinant MAP antigens, MAP2698c and MAP3567 were cloned, expressed and purified as previously described (Gurung et al., 2012b). Four mineral oil based MONTANIDE ISA adjuvants were used for the formulation of the recombinant vaccines. The adjuvants were 50V2, 71VG, and 61VG (water in oil formulations) and 201VG (a water in oil in water formulation). Each recombinant vaccine was formulated by mixing the required antigen with the selected adjuvants to obtain a 50 μg/ml final concentration of antigen. The required volume of recombinant vaccine was prepared by mixing antigen and adjuvant at 1:1 ratio (antigen + 50V2, antigen + 201VG); 2:3 ratio (antigen + 61VG) and 3:7 ratio (antigen + 71VG) and vortexed until emulsified.

### Immunization of animals

The sheep within the groups were vaccinated with: MAP2698c + 50V2 (group I), MAP2698c + 61VG (group II), MAP2698c + 71VG (group III), MAP2698c + 201VG (group IV), MAP3567 + 50V2 (group V), MAP3567 + 61VG (group VI), MAP3567 + 71VG (group VII), MAP3567 + 201VG (group VIII) (Table 1). The animals were given 1 ml of the required vaccine formulation by subcutaneous injection behind the left ear on the upper neck region. Four weeks after primary immunization, a booster was given on the right side of the neck with the same vaccine.

**Table 1:** **Number of animals with cellular (IFN-γ SP% > 38%) and humoral immune (antibody **SP%** > 70%) responses to recombinant antigen vaccine**.

Group^*^		**Number of animals responding to vaccine**			
	**Vaccine formulation**	**IFN-γ response**		**Antibody response**	
	**Antigen + adjuvant**	***a***	***b***	***a***	***b***
I	MAP2698c + 50V2	1	1	0	3
II	MAP2698c + 61VG	0	2	0	2
III	MAP2698c + 71VG	0	0	0	4
IV	MAP2698c + 201VG	0	3	0	2
V	MAP3567 + 50V2	1	1	4	4
VI	MAP3567 + 61VG	2	3	4	4
VII	MAP3567 + 71VG	0	0	3	4
VIII	MAP3567 + 201VG	1	0	3	4

a, MAP 316v specific response; b, vaccine antigen specific response;

* Four animals in each group.

### Blood sampling and injection site lesion monitoring

Blood was collected by venipuncture into lithium-heparin tubes (9 ml) and a tube without anti-coagulant (8 ml) (Vacuette®) on the day of primary vaccination (pre-vaccination) and at 2 week intervals thereafter (post-vaccination) except the final bleed which had only a 1 week interval. The injection site was inspected for lesions at the time of blood sample collection. The injection site areas were palpated and if a lesion was present, its size was recorded. A Vernier caliper was used to measure one directional lesion diameter in cm.

### IFN-gamma assay

#### Whole blood stimulation

Heparinized blood (500 μl per well) was placed in a 48-well plate (Falcon®) and stimulated with 500 μl of antigen at a required final concentration: 10 μg/ml of French pressed whole cell MAP strain 316v (MAP 316v) antigen, 5 μg/ml of pokeweed mitogen (PWM); and 10 μg/ml of MAP2698c or MAP3567 recombinant antigen. The 316v antigen was used for MAP-specific stimulation, PWM was used as a non-specific stimulant and recombinant MAP antigens were used for antigen-specific stimulation. All antigens were diluted in culture media containing RPMI 1640, 10% v/v fetal calf serum (FCS), penicillin, streptomycin, β-mercaptoethanol, and L-glutamine (GIBCO®, Life Technologies). The negative control consisted of 500 μl of culture medium with 500 μl of blood. The plate was incubated at 37°C with 5% CO_2_ for 48 h. The plasma supernatant was then harvested and stored at −20°C. All blood stimulation experiments were set up immediately after each bleed.

#### IFN-γ ELISA

The IFN-γ ELISA was performed as previously described (Wood et al., 1990; Begg et al., 2009). Optical density values were normalized across plates using the following calculation: Sample-to-positive (SP) % = [(Mean sample OD) − (Mean negative control OD)]/[(Mean positive control OD) − (Mean negative control OD)] × 100.

### Serum antibody assay

#### Serum adsorption

The serum was diluted (1:100) in a diluent (0.1% v/v FCS in PBS 0.05% v/v Tween 20) (FCS in PBST) containing 1.3 mg/ml of heat-killed *Mycobacterium phlei (M. phlei)* (Elizabeth Macarthur Agricultural Institute, New South Wales, Australia) and adsorbed overnight at 4°C with constant end-to-end shaking. The adsorbed serum was centrifuged at 2500 × *g* for 10 min at room temperature (RT) to separate the supernatant from the particulate *M*. *phlei*.

#### Serum antibody ELISA

The antibody ELISA was performed using a modified version of a previously described method (Yokomizo et al., 1983, 1985). Antigens (MAP 316v, recombinants MAP2698c and MAP3567) diluted in coating buffer (0.1 M carbonate buffer, pH 9.6) were immobilized onto flat-bottom 96-well microplates (Nunc MaxiSorp, South Australia, Australia) and incubated overnight at 4°C. The plate was machine-washed (Tecan, Aim Lab, Victoria, Australia) 5 times with wash buffer (reverse osmosis water with tween 0.05% v/v) and then blocked with 100 μl/well of a mixture of 1% v/v FCS (Gibco®, Victoria, Australia) at RT for 30 min.

The plate was machine washed 5 times as above. The adsorbed serum supernatant (50 μl) was added to the required wells and incubated for 1 h at RT. The plate was machine as above prior to the addition of horse radish peroxidase (HRP)-labeled mouse anti-sheep monoclonal conjugate (Clone GT-34, Sigma, New South Wales, Australia) (50 μl) (1:40,000) in diluent (0.1% v/v FCS in PBST), and then incubated for 1 h at RT. The plate was machine washed as above and 100 μl of 3′, 3′, 5′, 5′ tetra-methyl-benzidine (TMB) substrate was added. The plate was incubated at RT for 20 min in the dark after which the chromogenic reaction was stopped by the addition of stop solution (50 μl of 2 M sulphuric acid). The optical density (OD) was measured at 450 nm using a plate reader (Multiskan Ascent, Thermo Scientific, Victoria, Australia).

All serum samples were tested after each bleed. The ELISA result is presented as sample-to-positive (SP) % = [(Mean sample OD) − (Mean negative control OD)]/[(Mean positive control OD) − (Mean negative control OD)] × 100.

### Statistical analysis

Significant differences in IFN-γ and antibody responses between samples collected pre-vaccination and post vaccination were analyzed by one-way ANOVA with Bonferroni corrections for multiple comparisons in each vaccine group as previously described (Burton et al., 2009) using GraphPad Prism 4.0 (GraphPad Software Inc., La Jolla, USA) (*P* < 0.05, 95% CI). Responses were also compared between vaccinated and unvaccinated controls.

## Results

### IFN-γ response

**Figure 1:**
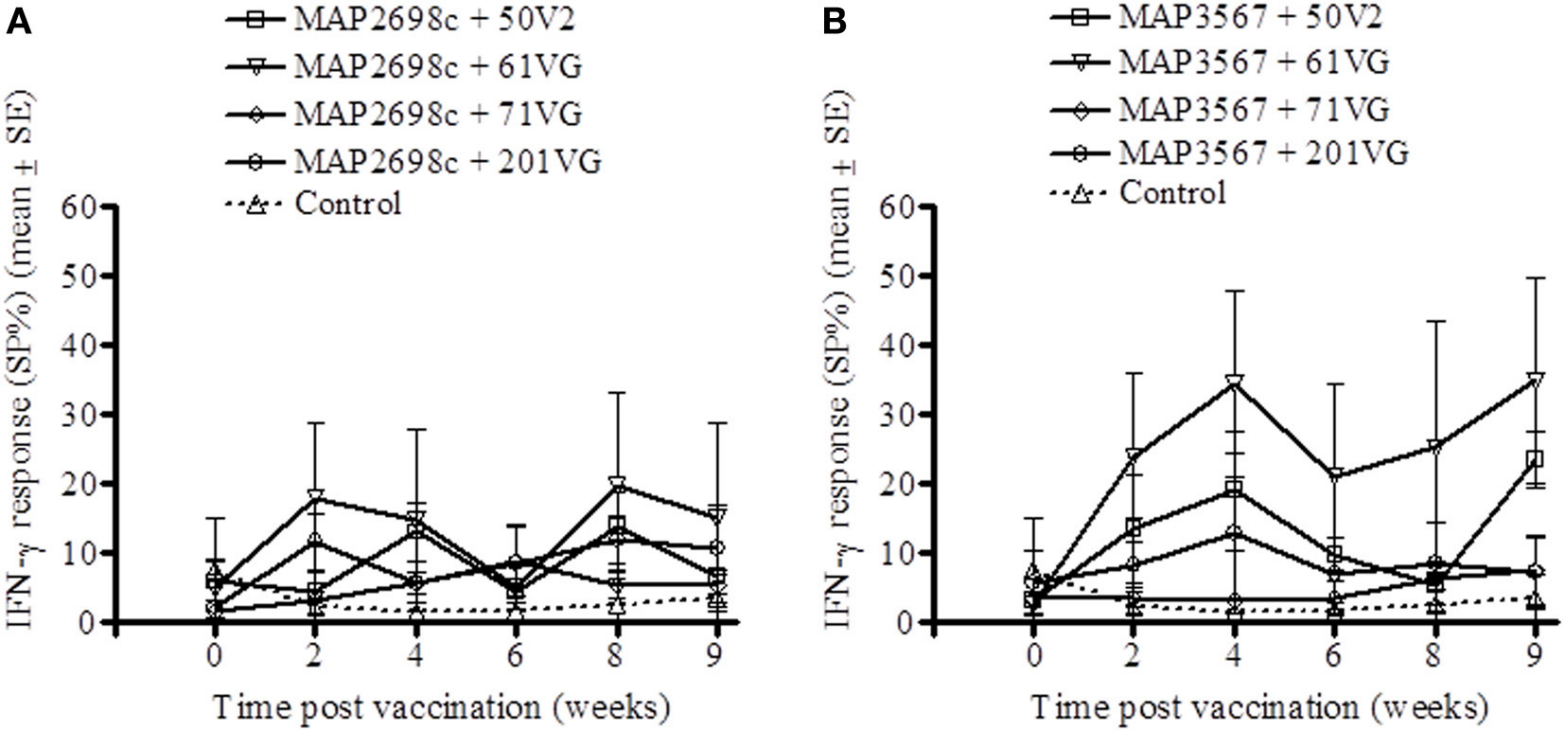
**MAP 316v specific IFN-γ responses from recombinant antigen vaccinated sheep. (A)** Responses in sheep vaccinated with MAP2698c vaccine; **(B)** Responses in sheep vaccinated with MAP3567 vaccine. Data are mean ± *SE*.

The cell mediated immune response to the recombinant vaccine was evaluated by measuring MAP 316v antigen as well as vaccine antigen specific IFN-γ responses. MAP 316v specific IFN-γ responses of the sheep in response to vaccination with the formulation of MAP2698c were similar between the adjuvants (Figure 1A). The sheep vaccinated with MAP3567 + 61VG formulation showed the highest MAP 316v specific IFN-γ responses among the different formulations (Figure 1B). However, great variation was observed between the groups of sheep vaccinated with MAP3567 vaccines depending on the adjuvant.

**Figure 2:**
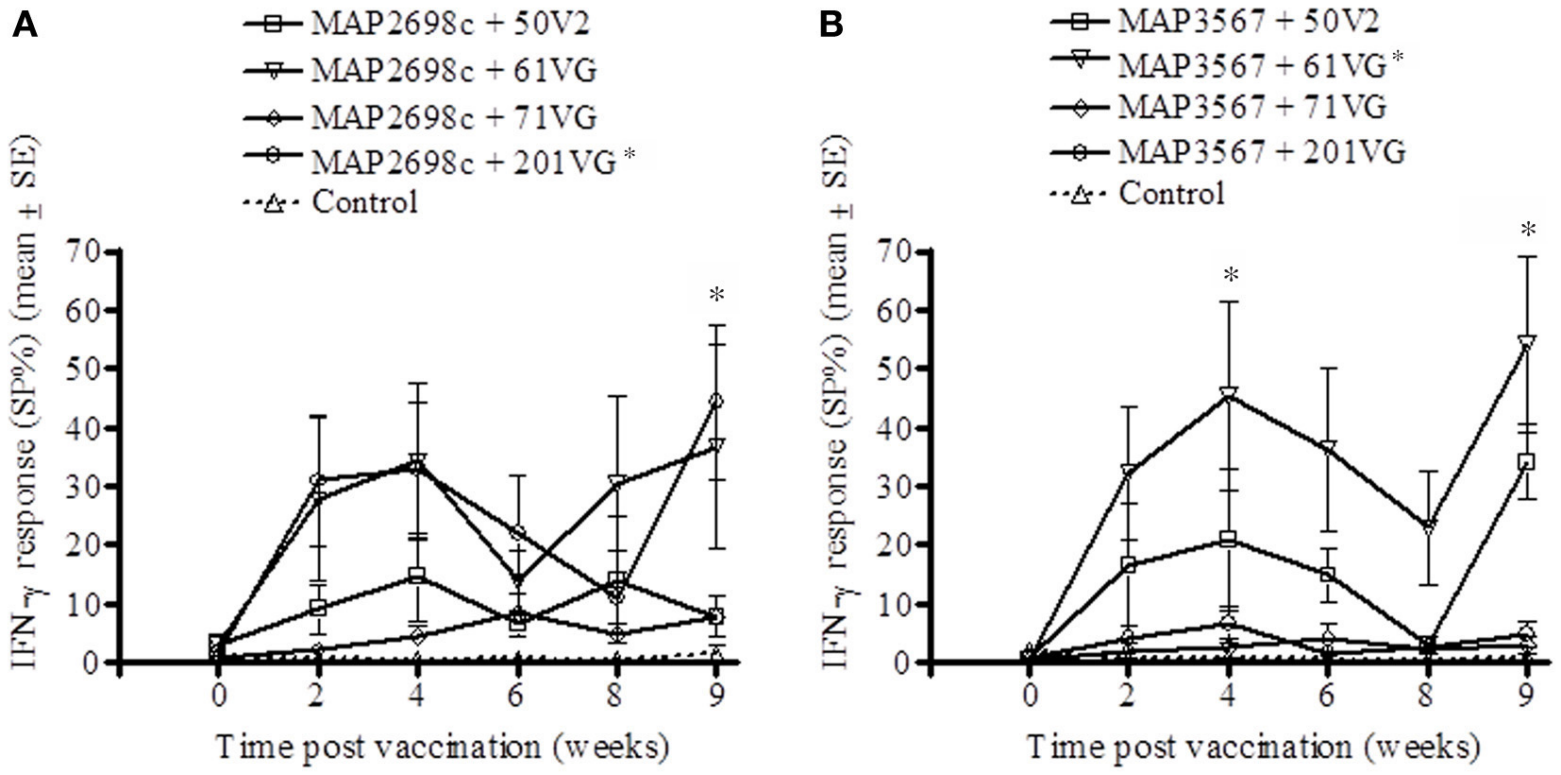
**Vaccine antigen specific IFN-γ responses of sheep vaccinated with the different formulations of recombinant antigen vaccines. (A)** MAP2698c-specific IFN-γ responses from sheep vaccinated with MAP2698c + adjuvants; **(B)** MAP3567-specific IFN-γ responses from sheep vaccinated with MAP3567 + adjuvants. Data are mean ± *SE*. ^*^Significantly higher SP% compared to pre-vaccination (*P* < 0.05).

The vaccine antigen (MAP2698c) specific IFN-γ response was highest for formulations prepared from MAP2698c + 61VG and MAP2698c + 201VG (Figure 2A) with the response to MAP2698c + 201VG formulation at 9 weeks post-vaccination significantly higher than those pre-vaccination (*P* < 0.05). Similarly, formulations prepared from MAP3567 + 61VG induced the highest vaccine antigen specific IFN-γ responses followed by the MAP3567 + 50V2 formulation (Figure 2B). The IFN-γ responses from MAP3567 + 61VG formulation at 4 and 9 weeks post-vaccination were significantly higher than those pre-vaccination (*P* < 0.05).

### Serum antibody response

**Figure 3:**
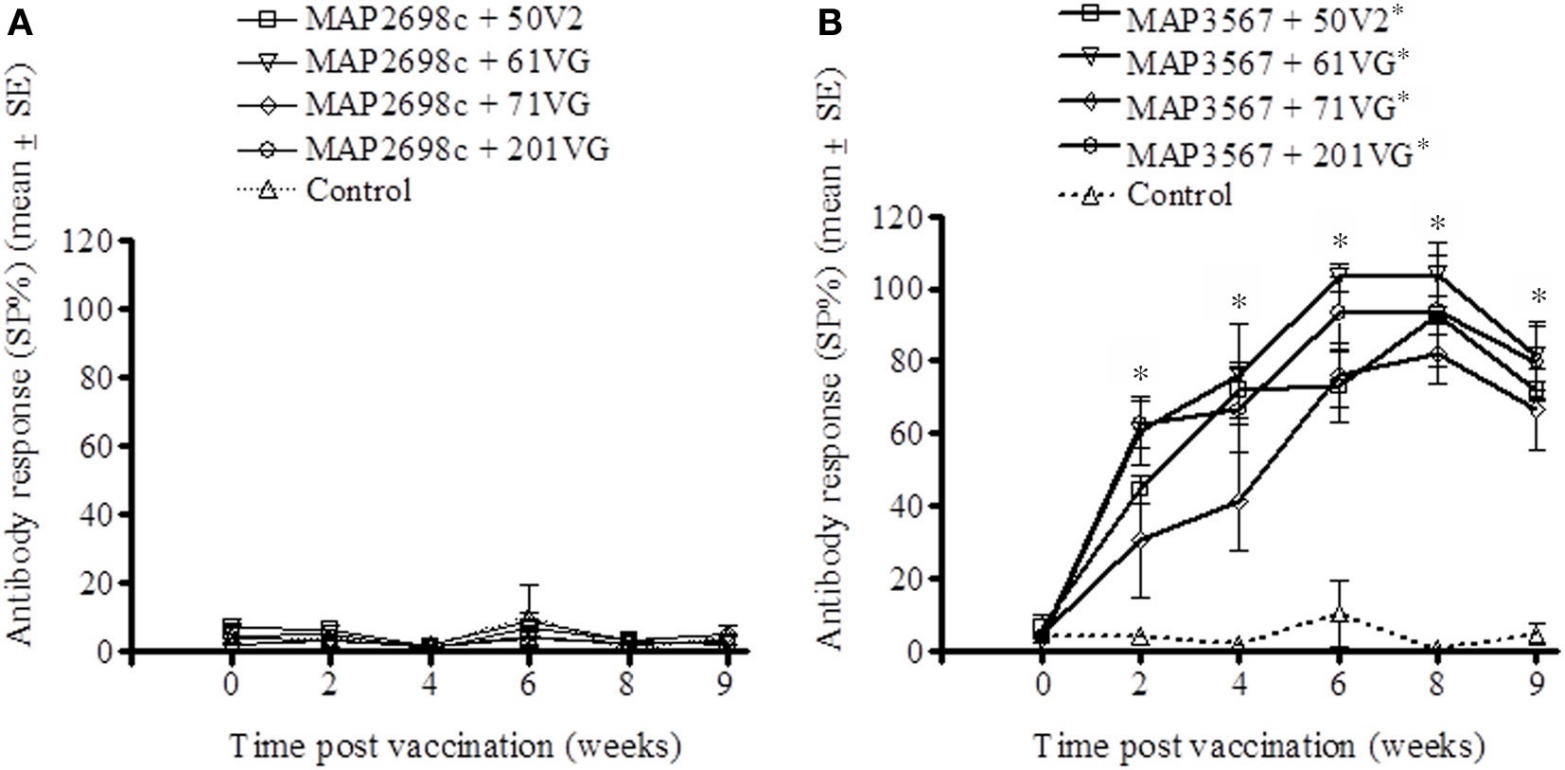
**MAP 316v antibody responses of serum from sheep vaccinated with the different formulations of recombinant antigen vaccines. (A)** MAP 316v antibody responses in sheep vaccinated with MAP2698c + adjuvants; **(B)** MAP 316v antibody responses in sheep vaccinated with MAP3567 + adjuvants. Data are mean ± *SE*. ^*^Significantly higher SP% compared to pre-vaccination (*P* < 0.05).

The humoral immune response to recombinant vaccine was evaluated by measuring MAP 316v and vaccine antigen specific serum antibody levels. MAP 316v antibody responses from sheep vaccinated with MAP2698c and all four adjuvant combinations were not distinguishable from those pre-vaccination or from unvaccinated controls (Figure 3A). The MAP 316v antibody responses from the vaccines formulated with MAP3567 and all four adjuvants showed significant responses (*P* < 0.05) which remained high until 8 weeks post primary vaccination (Figure 3B).

**Figure 4:**
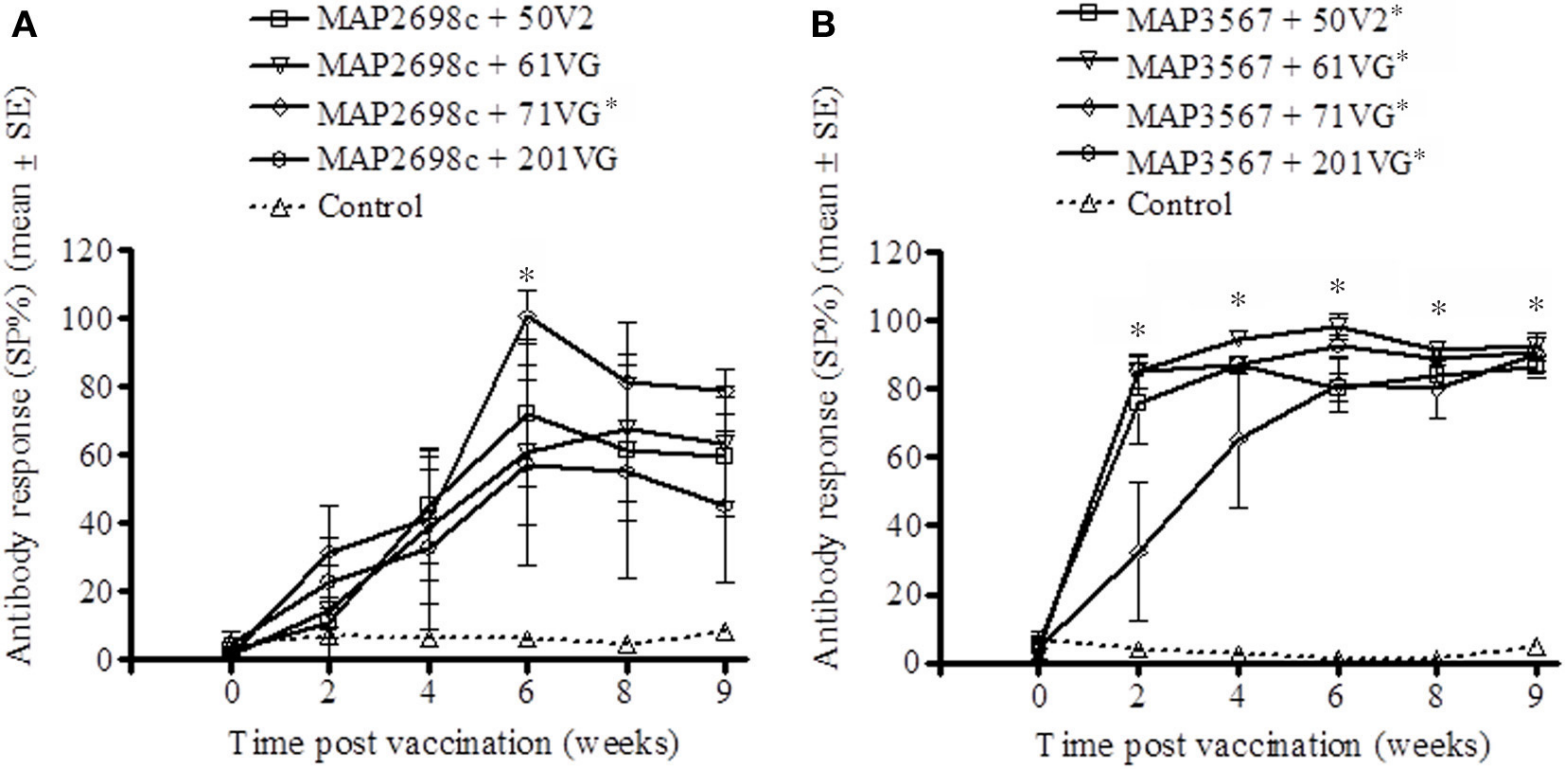
**Vaccine antigen specific antibody responses of serum from sheep vaccinated with the different formulations of recombinant antigen vaccines. (A)** MAP2698c-specific antibody responses in sheep vaccinated with MAP2698c + adjuvants; **(B)** MAP3567-specific antibody responses in sheep vaccinated with MAP3567 + adjuvants. Data are mean ± *SE*. ^*^Significantly higher SP% compared to pre-vaccination (*P* < 0.05).

The vaccine antigen specific antibody responses were strong for MAP2698c (Figure 4A) as well as MAP3567 (Figure 4B) vaccines. The formulations of MAP2698c with the four adjuvants showed similar patterns of vaccine antigen specific antibody responses, and the response to MAP2698c + 71VG at 6 weeks post primary vaccination was significantly higher than those pre-vaccination (*P* < 0.05). In contrast, antibody responses to MAP3567 formulations were similar to those of MAP 316v specific antibody responses. The antibody responses to the formulations of MAP3567 and all four adjuvants were significantly higher than those pre-vaccination (*P* < 0.05) at all sampling time-points (*P* < 0.05).

### Proportion of animals responding to vaccination

A recent study conducted to examine the protective level of IFN-γ response using a validated animal infection model (Begg et al., 2010) found that an 38% or greater SP of IFN-γ response was suggestive of a protective response in animals challenged with live MAP inoculum (de Silva., personal communication). A cut-point of 70% SP antibody as an indicator of humoral immune response to exposure was considered. These two thresholds (38% SP IFN-γ response and 70% SP antibody response) were used to examine the proportion of animals responding to recombinant vaccine. The number of animals with MAP 316v and vaccine antigen specific responses above these thresholds is shown in Table 1. Statistical analysis was not undertaken due to the small group sizes. A higher proportion of animals showed > 38% SP IFN-γ response to formulations prepared from MAP2698c + 61VG, MAP2698c + 201VG, and MAP3567 + 61VG (range: 39–90 SP%). The proportion of animals showing vaccine antigen specific IFN-γ responses was higher than that of MAP 316v specific responses. None of the animals that received MAP2698c vaccine formulations showed MAP 316v specific antibody responses > 70% SP.

### Injection site lesions and lesion prevalence

**Figure 5:**
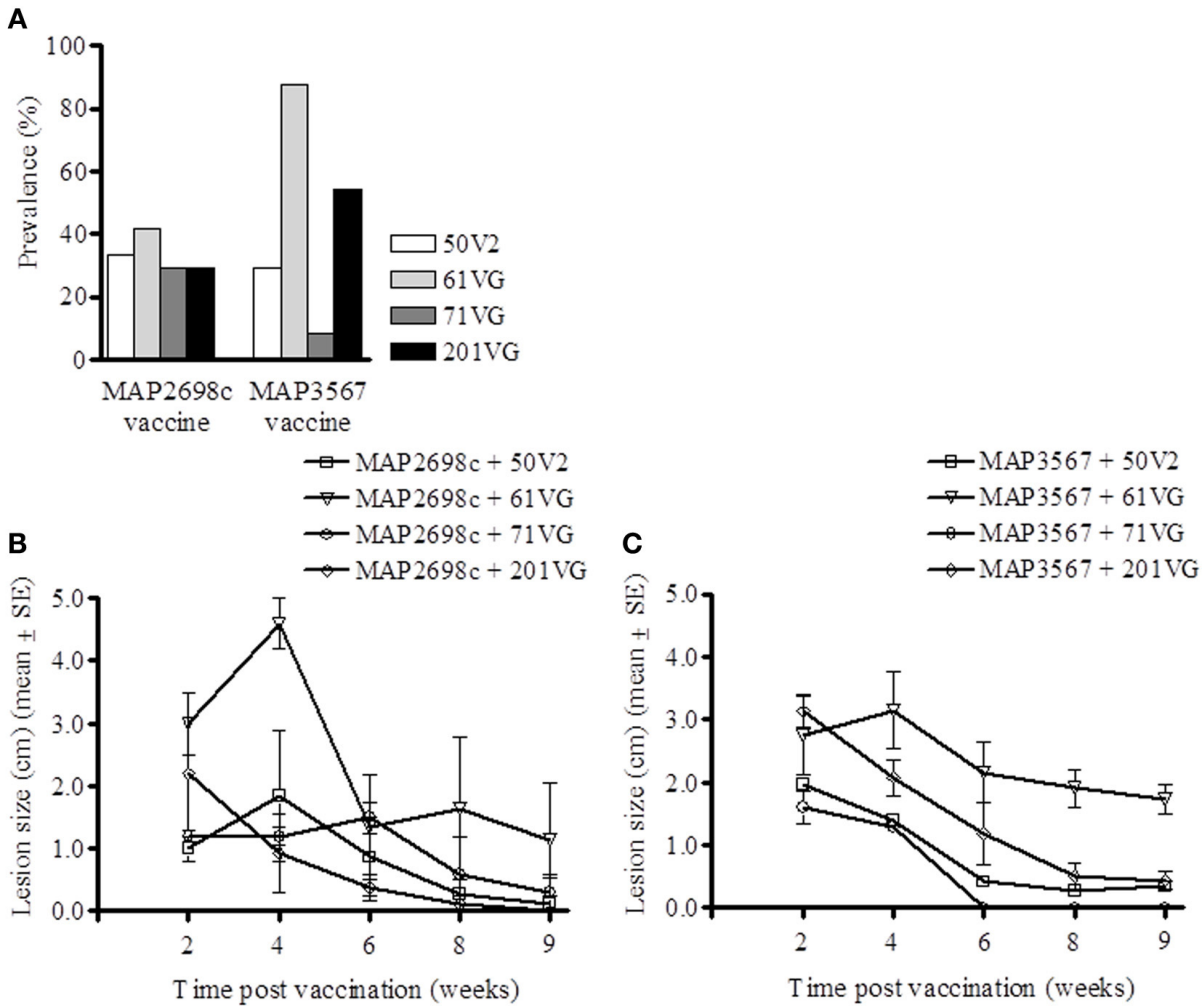
**Vaccine injection site lesion prevalence and lesion size. (A)** Lesion prevalence during the study period; **(B)** Vaccine formulation (MAP2698c + adjuvants) lesion size and trend; and **(C)** Vaccine formulation (MAP3567 + adjuvants) lesion size and trend. Data are mean ± *SE*.

Lesion prevalence was analyzed for each antigen/adjuvant combination. The majority of the animals (75%) developed injection site lesions i.e., lesion diameter > 0.5 cm. The animals that developed the most lesions were those vaccinated with MAP3567 + 61VG (87.5%) and MAP3567 + 201VG (54.2%) as shown in Figure 5A. Average lesion prevalence in animals vaccinated with MAP2698c (33%) was lower than in those vaccinated with the MAP3567 vaccines (45%).

Except for the animals vaccinated with MAP2698c + 61VG, the mean lesion size for all other adjuvant groups were smaller than 2.2 cm and decreased over the study period (Figure 5B). The mean lesion size for MAP2698c + 61VG was 2.35 cm and rapidly decreased over the study period. In animals that received MAP2698c + 201VG formulation, the injection site lesions had completely resolved by the end of the study period. The injection site lesion recovery in animals that received MAP3567 vaccine was similar between different formulations (Figure 5C). However, the lesions that developed in response to the MAP3567 vaccine were more severe in comparison to those in response to MAP2698c vaccine. The mean lesion size was greatest for the MAP3567 + 61VG group and persisted at 1.73 cm until the end of the study period. Some of these lesions resulted in wool loss and a discharging sinus at the injection sites in animals vaccinated with MAP3567 + 61VG. A higher proportion of animals vaccinated with vaccine MAP3567 had large lesions compared to those vaccinated with MAP2698c.

## Discussion

The evaluation of recombinant MAP antigens as vaccine candidates in this study was focused on cell mediated and humoral immune responses in sheep following vaccination with different formulations prepared from combinations of recombinant antigens and four different adjuvants. Expression of cytokines such as IFN-γ is believed to contribute to protection against intracellular pathogens including MAP but it is not known what level of response is adequately protective (Appelberg, 1994; Appelberg et al., 1994; Stabel, 1996; Chandra et al., 2012). A recent study using a validated animal infection model has suggested that animals with an early IFN-γ response of 38% SP or greater are more likely to be protected against oral challenge with live MAP (de Silva et al., personal communication). This cut-off was used in this study to assess the proportion of sheep with an IFN-γ response. At worst it can be regarded as an arbitrary cut-off. In this study the recombinant vaccine formulations were found to induce strong IFN-γ responses, as high as 90% SP, in some vaccinated animals. The assessment of the best vaccine formulation recommneded to carry forward from this study was based on five criteria: (a) antigen specific IFN-γ response, (b) MAP 316v specific IFN-γ response, (c) adjuvant effect on IFN-γ response, (d) injection site lesions and (e) antibody response. Recombinant vaccine formulated with MAP2698c antigen and 201VG adjuvant induced stronger MAP 316v as well as recombinant antigen specific IFN-γ responses in vaccinated animals compared to the unvaccinated controls and pre-vaccination. The vaccine also produced lower lesion prevalence and severity compared to other formulations. The antibody response was slightly lower from this formulation, and antibody is thought to play a lesser role in protection against MAP infections (Rosseels and Huygen, 2008).

Lesions at the vaccine injection site have been observed in a high proportion of animals following vaccination against paratuberculosis (Sigurdsson and Tryggvadottir, 1950; Chiodini et al., 1984; Windsor and Eppleston, 2006). Lesions are thought to be caused by the oil-based adjuvants which are non-absorbable, act as irritants and increase antigen persistence to induce sustained immune responses (Hope, 1995). In lambs that received Gudair™ vaccine, 65% of them developed injection site lesions and these lesions persisted for up to 4 years in 20% of the vaccinated animals (Eppleston and Windsor, 2007). The lesion prevalence and sizes in this study were monitored for a 9 week period following primary vaccination and were observed to be decreasing post-vaccination. The formulation MAP3567 and the adjuvant MONTANIDE™ ISA 61VG resulted in a high proportion of sheep with severe lesions. This formulation may not qualify as a vaccine candidate on animal welfare grounds due to the initial high number of large lesions and the possibility of downgrading carcass value at slaughter. These data also indicate that it is not just the adjuvants that are responsible for the injection site lesions but the combination between adjuvant and antigen. Sheep vaccinated with the MAP2698c formulation showed substantial levels of IFN-γ responses in a high proportion of vaccinated animals and low prevalence of injection site lesions. The vaccine formulated with MAP3567 showed a strong antibody response for all adjuvant groups and caused more severe lesions.

The findings from this study suggest that MAP2698c and MAP3567 antigens may have potential as candidate antigens for cell mediated and antibody mediated immune response studies, respectively. These findings are also supported by a previous study in which we reported the presence of a greater number of T cell epitopes for the MAP2698c protein and a greater number of B cell epitopes for the MAP3567 protein compared to other proteins (Gurung et al., 2012a). Antigenicity of these antigen was confirmed in clinically infected sheep Gurung et al. (2012b). Furthermore, MAP3567 is reported to be a surface-exposed protein overlapped with the cell wall proteins that may be more accessible to circulating antibodies and effect antigen-antibody binding to clear pathogens in natural infection conditions (He and De Buck, 2010). Sheep vaccinated with MAP2698c antigen were not able to produce MAP 316v specific antibodies. However, we previously reported that MAP2698c antigen is able to be used to detect specific antibodies from MAP infected sheep (Gurung et al., 2012b). These findings suggest that the MAP 316v antigen may either not contain MAP2698c protein or it is not in a form that can be recognized by the serum antibodies from MAP2698c vaccinated sheep.

Due to the likely protective immune function of IFN-γ and the relatively lower degree of lesion development at the site of vaccine injection from MAP2698c + 201VG vaccine compared to that of other formulations, this vaccine should be evaluated further in sheep. A longitudinal study in a larger cohort of animal is required to evaluate whether it induces protective immunity against MAP infection.

### Conflict of interest statement

This work was supported by Meat and Livestock Australia and by Cattle Council of Australia, Sheepmeat Council of Australia and WoolProducers Australia through Animal Health Australia.
